# Urine Biomarkers Combined With Ultrasound for the Diagnosis of Obstruction in Pediatric Hydronephrosis

**DOI:** 10.3389/fped.2021.762417

**Published:** 2022-01-06

**Authors:** Vytis Kazlauskas, Vytautas Bilius, Virginijus Jakutis, Renata Komiagiene, Birute Burnyte, Gilvydas Verkauskas

**Affiliations:** ^1^Clinic of Gastroenterology, Nephrourology and Surgery, Institute of Clinical Medicine, Faculty of Medicine, Vilnius University, Vilnius, Lithuania; ^2^Clinic of Anesthesiology and Intensive Care, Institute of Clinical Medicine, Faculty of Medicine, Vilnius University, Vilnius, Lithuania; ^3^Department of Radiology, Nuclear Medicine and Medical Physis, Institute of Biomedical Sciences, Faculty of Medicine, Vilnius University, Vilnius, Lithuania; ^4^Department of Human and Clinical Genetics, Institute of Biomedical Sciences, Faculty of Medicine, Vilnius University, Vilnius, Lithuania

**Keywords:** hydronephrosis, ureteropelvic junction obstruction, urine biomarkers, renal scan, ultrasound

## Abstract

**Introduction:** To establish the efficacy of ultrasound (US) combined with urine biomarkers in differentiating patients who require surgical management from those who do not, avoiding invasive investigations.

**Materials and Methods:** From February 2019 to February 2021, all pediatric patients who presented with hydronephrosis were selected for the study. All renal units (RU) were evaluated by US, and fresh frozen voided urine samples were collected at the time of inclusion. Hydronephrosis grade was evaluated by the Society for Fetal Urology (SFU) and an alternative grading system (AGS). Patients who had high-grade hydronephrosis on US were referred to renal scan (RS) or intervention, when there was an increase of dilatation in subsequent follow-up images. Fresh frozen urine from the control group with no history of renal diseases and no renal anomalies on US was collected. We compared differences of US parameters combined with urine biomarkers between surgically and non-surgically managed patients and between the groups of patients when they were stratified by different RS findings and analyzed whether urinary biomarkers give any additional value to US. Instead of the anterior–posterior diameter (APD), we used its ratio with mid-parenchymal thickness. The additional efficacy of biomarkers to US was calculated when the US component was derived to a cumulative APD/mid-parenchymal ratio.

**Results:** Sixty-four patients with hydronephrosis were prospectively included in the study accounting for a total of 81 patient visits and 162 RUs evaluated. A control group of 26 patients was collected. The mean age at inclusion in the hydronephrosis group was 43.7(±45.5) months, and a mean age in a control group was 61.2(±41.3) months. The cumulative APD/mid-parenchymal ratio combined with urinary albumin, β2 microglobulin (β2-M), and urinary neutrophil gelatinase-associated lipocalcin may have a better performance in the prediction of surgical intervention than the cumulative APD/mid-parenchymal ratio alone (*p* = 0.1). The best performance to detect the increased tissue transit time and obstructive curve on RS was demonstrated by the β2-M creatinine ratio. An increased cumulative APD/mid-parenchymal ratio with biomarkers together had a fairly good sensitivity and specificity for detection of DRF < 40%.

**Conclusions:** According to our data, the APD/mid-parenchymal ratio alone has good efficacy in prediction of surgery and abnormal RS findings especially when combined with urine biomarkers.

## Introduction

Congenital hydronephrosis is one of the most common birth anomalies requiring close follow-up during the first years of life and even further in childhood (1, 2). Most of the cases are self-limiting and cause no harm to the kidneys, but about 20% show split renal function deterioration (3). The main diagnostic facility is ultrasound (US), which enables to define the stage of hydronephrosis and the need of further evaluation (4). A single US scan alone has inconsistent diagnostic value, and it could not predict the renal function and drainage pattern especially in high-grade hydronephrosis (5, 6). Delayed surgery when significant obstruction is present seems to give worse results than early procedure (7, 8). For this reason, early diagnosis and decision making are desirable. Renal scan (RS) and magnetic resonance urography are recognized tools to support the diagnosis of obstruction; however, these diagnostic modalities are invasive, emit radiation, reduce cost-effectiveness, or require general anesthesia (9). Unfortunately, obstruction is so far best defined by Koff as “any restriction to urinary flow which, if left untreated, would cause damage to the kidney” (10). Most probably, the development of obstruction is a continuous process in close connection with developing diuresis, kidney morphology, and surrounding anatomy. Therefore, dynamic evaluation is so important. Development of new harmless and clinically optimized diagnostic tools remains important.

These diagnostic modalities have been discussed recently. Onen's alternative grading system combined with Doppler evaluation of ureteral jets, renal parenchyma to hydronephrosis area ratio, and US-based scoring system for pyeloplasty were suggested as single tools to evaluate congenital hydronephrosis for obstruction and risk of pyeloplasty and appeared to have a better performance than anterior–posterior diameter (APD), Society for Fetal Urology (SFU) grade, or urinary tract dilation only (11–13). Another significant step toward non-invasive insight into hydronephrosis is a discovery of new urine biomarkers. Urine proteome analysis demonstrated elevated concentrations of 50–80 proteins in bladder urine when obstructed kidney was present, compared to controls (14–16). A recent systematic review pointed out the most frequent proteins described as potential markers of obstruction. Urinary neutrophil gelatinase-associated lipocalin (uNGAL), monocyte chemotactic peptide-1, transforming growth factor β1, epidermal growth factor, and kidney injury molecule 1 (KIM 1) were associated with increased risk of obstructive uropathy (17).

The aim of our study was to elucidate whether most ordinary and cost-effective urinary biomarkers of kidney injury give any additional value to US to improve its performance in the prediction of obstruction on RS, increased tissue transit time (TTT), low differential renal function (DRF), and choice of surgery.

## Materials and Methods

From February 2019 to February 2021, all pediatric patients who presented with hydronephrosis were prospectively included in the study. The criteria for exclusion were single kidney, contralateral kidney aplasia/dysplasia, acquired obstruction, patients older than 12 years, and parental refuse. All renal units (RU) were evaluated by renal US, and fresh frozen voided urine samples were collected at the time of inclusion and in part of the patients at the second appointment. Hydronephrosis grade was evaluated by SFU and AGS systems. Renal volume, APD, the largest caliceal diameter, and mid-parenchymal thickness were recorded. US evaluation in all patients was done by a single person. For US scans, we used a LOGIQ V2 portable US device. Patients who had high-grade hydronephrosis (SFU 3–4 and AGS 3–4) on US were referred to RS. Surgery was undertaken when signs of obstruction were present on examination. According to the local protocol, obstruction is diagnosed and surgery indicated when there is an increase of dilatation in subsequent follow-up images by 20% of APD diameter, differential function below 40%, or symptoms (pain, pyelonephritis). Following this protocol, signs of obstruction were confirmed by the operative findings in all operated patients.

RS was done using the Infinia2 dual detector imaging system. The 99mTc MAG3 radionuclide was used following F-20 protocol in a suspicion of obstruction on renogram. DRF, curve pattern, and TTT were evaluated. One second/frame for the first 2 min and 15 s/frame for the remaining 40 min were used to assess TTT. TTT was assumed as the time from the first sign of radionuclide in renal parenchyma until the first sign of radionuclide in the renal collecting system. TTT was assessed by a single radiologist. Fresh frozen urine from a control group who had no history of renal diseases and no renal anomalies on US was collected. Urine samples in all groups were obtained using urine collection bags in small children and older children voided on demand directly into the urine collection container. Urine samples in 1–2 h were centrifuged and frozen at −80°C. When the whole batch was collected, urinary albumin (uAlb), urinary β2 microglobulin (β2-M), and urinary uNGAL concentrations and their creatinine ratios were measured in all groups. For biochemical analysis, we used the Abbot Architect analyzer. First of all, we selected the best US predictor for surgical intervention, then compared its performance between the respective groups when combined with biochemical urine markers. We also aimed at defining whether US with urine biomarkers is efficient enough to determine any of the RS parameters (DRF < 40%, TTT > 3 min, and obstructive curve classified as B or D by O'Reilly's classification). Logistic regression was used to detect significant prognostic factors for surgical management and previously defined RS parameters. Receiver operating characteristic (ROC) curves were generated to reveal whether there was any significant improvement in diagnostic ability when US was combined with biochemical markers. Instead of broadly using APD, we used its ratio with mid-parenchymal thickness which was significant for the prediction of operation. The efficacy of biomarkers to US was calculated when the US component was derived to a cumulative APD/mid-parenchymal ratio (sum of APD/mid-parenchymal ratios of both kidneys) assuming that urine biomarkers may be influenced by both RUs regardless of unilateral or bilateral hydronephrotic involvement. Kaplan–Meier survival analysis was employed to demonstrate the proportion of operated vs. non-operated patients when the model suggested different outcomes.

This study was approved by the Vilnius Regional Bioethics Committee (158200-18/6-1044-544, the date of approval 05/06/2018). For calculation, we used R version 4.1.0.

## Results

Sixty-four (39 boys and 25 girls) patients with hydronephrosis were prospectively included in the study; 17 of them had repeated appointment, accounting for a total of 81 patient visits and 162 RU evaluations. A control group of 26 patients (23 boys and 3 girls) was collected. The mean age at inclusion in the hydronephrosis group was 43.7(±45.5) months and 61.2(±41.3) months in the control group. Unilateral hydronephrosis was present in 56 patients and bilateral involvement in 8. The median time of follow-up from the inclusion was 8.05 (2.3; 15.7) months. Thirty-six patients had left hydronephrosis, 26 right, and 8 bilateral. Forty patients were referred to RS comprising a total of 80 RU scanned.

**Figure 1:**
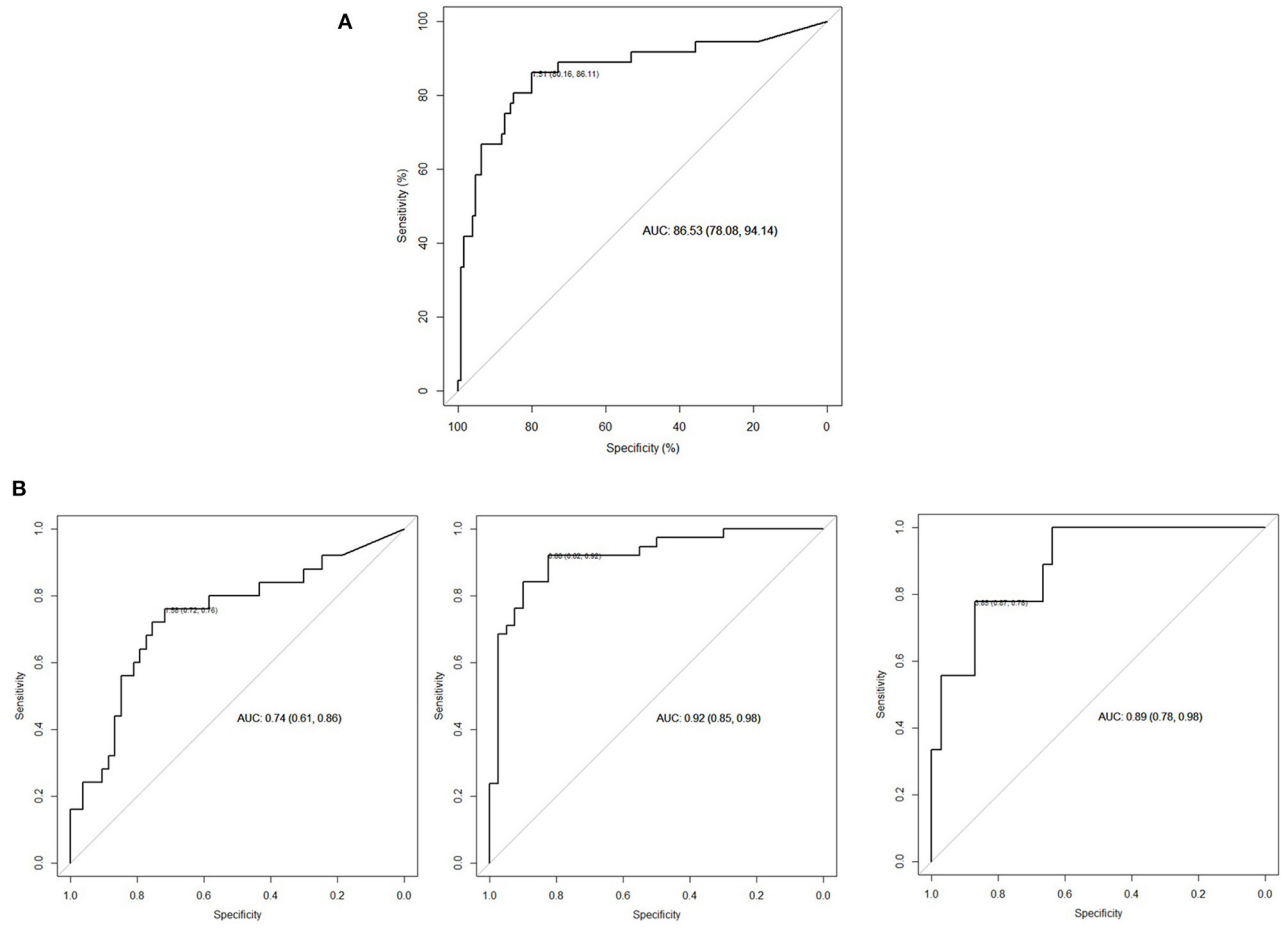
**(A)** ROC curve APD/mid-parenchymal ratio with a cutoff value for operation of 1.51 (*p* < 0.001). The odds ratio for operation when the APD/mid-parenchymal ratio is more than the cutoff (95% CI) is 1.97 (1.54; 2.51). *N* = 162 (RUs). **(B)** From left to right: diagnostic performance of the APD/mid-parenchymal ratio to detect TTT >3 min, cutoff 1.38; APD/mid-parenchymal ratio to detect obstructive curve (B, D types of o'Reilly's classification), cutoff 0.88; and APD/mid-parenchymal thickness to detect DFR < 40%, cutoff 3.85. *N* = 80 (RUs).

Logistic regression model and stepwise model selection demonstrated that significant US parameters for the prediction of operation were mid-parenchymal thickness and APD of the renal pelvis, while calyceal dilatation, renal volume, AGS, and SFU grade were not. Instead of two separate significant parameters, we used the APD/mid-parenchymal ratio, which was also a significant predicting factor for operation when RU were evaluated separately in all appointments (Figure 1A; Table 1) and was independent of age when compared between 0–4-, 5–8-, and 9–12-year groups (Kruskal–Wallis test for equal medians, *n* = 162, *p* = 0.68). ROC curve analysis demonstrated the APD/mid-parenchymal ratio providing a significant area under the curve (AUC) when utilized for prediction of TTT > 3 min, obstructive curve on renogram, and DRF < 40% for each RU (Figure 1B; Table 1). When the APD/mid-parenchymal ratio was combined with TTT (an early sign of renal damage) and compared to the APD/mid-parenchymal ratio alone in the prediction of surgery, the AUC did not differ and was equal to 0.88 (*p* = 1). The value of the APD/mid-parenchymal ratio in the control group of each single kidney (which was assumed as a normal group) was 0.25(±0.26) and ranged from 0 (in 22 RUs out of 52 available) to 0.68.

**Table 1:** Statistical values of the APD/mid-parenchymal ratio for the prediction of RS parameters and surgical intervention for each renal unit (*n* = 162).

***N*** = 80^*^ (RUs)	TTT > 3 min^*^	Obstructive curve^*^	DRF < 40%^*^	Surgical intervention^**^
***N*** = 162^**^ (RUs)				
**Measures**		**Point estimates and 95% CIs**		
SS	0.36 (0.18, 0.57)	0.76 (0.60, 0.89)	0.33 (0.07, 0.70)	0.47 (0.30, 0.65)
SP	0.87 (0.75, 0.95)	0.90 (0.76, 0.97)	0.99 (0.92, 1.00)	0.95 (0.90, 0.98)
PPV	0.56 (0.30, 0.80)	0.88 (0.72, 0.97)	0.75 (0.19, 0.99)	0.74 (0.52, 0.90)
NPV	0.74 (0.62, 0.84)	0.80 (0.65, 0.90)	0.92 (0.83, 0.97)	0.86 (0.79, 0.92)
PLR	2.73 (1.15, 6.48)	7.63 (2.96, 19.66)	23.00 (2.67, 198.23)	9.92 (4.22, 23.29)
NPL	0.74 (0.54, 1.01)	0.26 (0.15, 0.47)	0.68 (0.43, 1.07)	0.55 (0.41, 0.76)

*SS, sensitivity; SP, specificity; PPV, positive predictive value; NPV, negative predictive value; PLR, positive likelihood ratio; NLR, negative likelihood ratio*.

* *Data were calculated using the sample size of N = 80*,

** *Data was calculate using sample size N = 162*.

To check for age influence, the medians of concentrations of biomarkers in voided urine were compared between the respective age groups and no significant difference was found (*p* = 0.22 for uAlb, *p* = 0.49 for β2-M, and *p* = 0.38 for uNGAL). For the assessment of surgical intervention risk in each patient, we used the sum of APD/mid-parenchymal (cumulative) ratios of both kidneys and biomarkers in voided urine. The ROC curve analysis for the cumulative APD/mid-parenchymal ratio of both kidneys of surgical intervention on at least one RU during the follow-up was a significant predicting factor with a cutoff value of 3.32. The cumulative APD/mid-parenchymal thickness in a control group was 0.50(±0.39) and ranged from 0 (in 7 out of 26 pts) to 1.3

Biochemical factors altogether in the logistic regression model increased the AUC of the cumulative APD/mid-parenchymal ratio in the prediction of surgical intervention (*p* = 0.1) although insignificant. When patients were stratified by age into two groups below 6 years of age (n1 = 58) and after 6 years of age (n2 = 23), the respective AUC when the APD/mid-parenchymal ratio alone was compared to the APD/mid-parenchymal ratio with biomarkers for the prediction of surgery did not differ (<6 years, *p* = 0.2, and >6 years, *p* = 0.23). The diagnostic performance of uAlb alone compared to the uAlb/creatinine ratio demonstrated better performance of uAlb alone for surgical intervention (*p* = 0.02), cutoff 11.5 mg/l. Neither β2-M alone or β2-M/creatinine ratio nor uNGAL alone or uNGAL/creatinine ratio were significant predictors of operation (*N* = 81)

Differences of TTT, urine biomarkers, and cumulative APD/mid-parenchymal ratio between groups when patients were stratified by age are shown in Table 2.

**Table 2:** The utility of TTT, urinary biomarkers, and cumulative APD/mid-parenchymal thickness ratio between operated and non-operated patients in different age groups.

***N*** **= 81 pts**	**TTT of at least one kidney >3 min^‡^**		**uAlb (mg/l)**		**uAlb/crea (mg/mmol)**		**β2-M (mg/l)**		**β2-M/crea (mg/mmol)**		**uNGAL (ng/ml)**		**uNGAL/crea (μg/ml)**		**Cumulative APD/mid-parenchymal ratio**	
**Medians**																
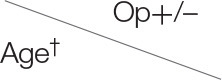	+	–	+	–	+	–	+	–	+	–	+	–	+	–	+	–
<6 years (n1 = 58) Op-21, non-op-37	12 (14)	4 (11)	11	3	3.26	2.72	0.09	0.04	0.055	0.044	4.2	4.4	1.93	4.31	4.37	1.94
*p* value			0.000456^*^		0.0484^*^		0.003904^*^		0.34		0.6714		0.1547		<0.001^*^	
>6 years (n2 = 23) Op-13, non-op-10	3(8)	1 (6)	8	8	1.2	1.15	0.05	0.06	0.01	0.012	2.9	2.0	0.63	0.48	5.02	1.9
*p* value	0.052	0.6	0.8761		0.5629		0.4461		0.6049		0.3948		0.6709		0.01471^*^	

*Medians compared by Wilcoxon signed-rank test*.

*^†^Age in years*.

*Op+/– indicates parameters for operated and non-operated patients and each p value below and in between them*.

‡ *The distribution of increased TTT was compared between >6- and <6-year-old groups in both operated and non-operated patients (Fisher's exact test) and p values below each column. A complete number of patients in each group in parentheses*.

* *Statistically significant value*.

**Figure 2:**
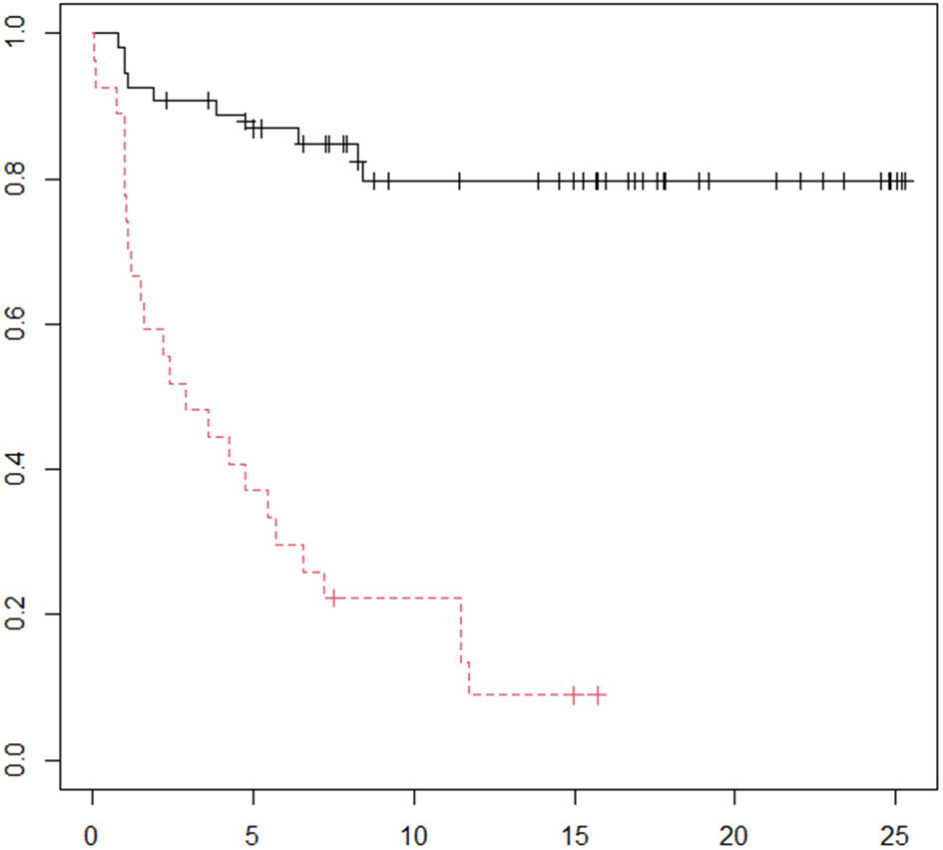
Kaplan–Meier curve for the time until operation. Red curve represents time when the model predicted the operation. Follow-up in the horizontal axis in months; *p* = 0.00004 (logRank). *N* = 81.

**Figure 3:**
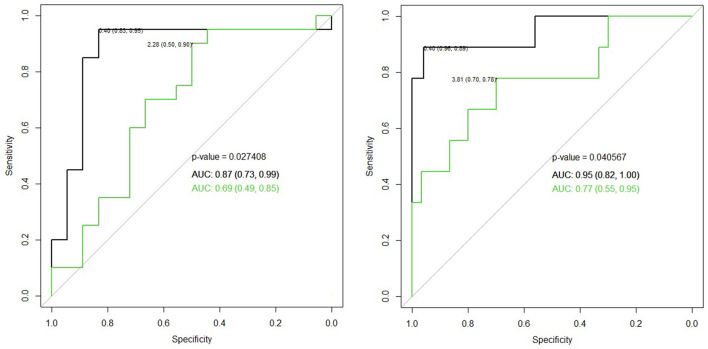
Left ROC curve for the cumulative APD/mid-parenchymal ratio alone (green) in comparison to the β2-M/creatinine ratio with a cumulative APD/mid-parenchymal ratio altogether in the logistic regression model (black) for the detection of TTT > 3 min of at least one RU, *p* = 0.036, cutoff value 0.03 of the β2-M creatinine ratio, and 2.28 of the cumulative APD/mid-parenchymal ratio. Right ROC curve for the cumulative APD/mid-parenchymal ratio (green) in comparison to β2-M, uNGAL, uAlb, and cumulative APD/mid-parenchymal ratio altogether in the logistic regression model (black) with bigger AUC of DRF < 40% of at least one RU, *p* = 0.04; cutoff values of the cumulative APD/mid-parenchymal ratio 3.81, β2-M 0.04 mg/l, NGAL 0.35 ng/ml, and uAlb 4.3 mg/l. *N* = 40.

Table 3 demonstrates the performance of the cumulative APD/mid-parenchymal ratio and its combination with biomarkers for the choice of surgical treatment. Comparisons of Kaplan–Meier survival curves until surgical intervention for the positive and negative prediction of model are demonstrated in Figure 2. For the prediction of RS parameters, we composed the multivariable logistic regression model for each condition: at least one kidney had increased TTT > 3 min; at least one obstructive curve; at least one T1/2 > 20 min, and a kidney with DRF < 40%. There were no significant independent variables for the curve pattern and T1/2. uNGAL and uAlb were not significant in the prediction of increased TTT, and the β2-M/creatinine ratio was significant. The best performance was demonstrated by the β2-M/creatinine ratio combined with a cumulative APD/mid-parenchymal ratio for TTT. The cumulative APD/mid-parenchymal ratio for DRF showed that AUC was bigger when the cumulative APD/mid-parenchymal ratio was combined with biochemical factors (Figure 3). Comparisons of uAlb, uNGAL, and β2-M concentrations in voided urine between the surgical intervention, non-surgical follow-up, and control groups and their normal values are provided in Table 4 (18–20). The urine concentrations of biomarkers in a relation to TTT and curve pattern are demonstrated in Table 5.

**Table 3:** Statistical evaluation of the cumulative APD/mid-parenchymal ratio of both kidneys and biochemical bladder urine factors for the prediction of surgical intervention in at least one RU.

***N*** **= 81**	**Cumulative APD/mid-parenchymal ratio**	**Cumulative APD/mid-parenchymal ration with β2-M, uAlb, uNGAL**
**Measures**	**Point estimates and 95 % CIs**	
SS	0.67 (0.52, 0.83)	0.71 (0.55, 0.86)
SP	0.89 (0.80, 0.98)	0.94 (0.87, 1)
PPV	0.82 (0.68, 0.96)	0.89 (0.77, 0.1)
NPV	0.79 (0.68, 0.90)	0.81 (0.71, 0.91)
PLR	6.35 (2.64, 15.22)	11.06 (4.21, 28.96)
NPL	0.36 (0.23, 0.54)	0.31 (0.19, 0.55)

**Table 4:** Kruskal–Wallis test to compare medians of biochemical factors in voided urine between the surgically managed, follow-up, and control groups.

***N*** **= 90 medians**	**Follow-up** **(***n*** = 46)**	**Surgical intervention** **(***n*** = 35)**	**Control** **(***n*** = 26)**	***p*** **value**
uAlb (mg/l)	4.0	8.5	6.0	0.002922^*^
uAlb/creatinine (mg/mmol)	1.92	3.17	1.58	0.09669^†^
β2-M (mg/l)	0.04	0.07	0.08	0.04043^*^
β2-M/creatinine (mg/mmol)	0.03	0.03	0.016	0.06247^†^
uNGAL (ng/ml)	3.1	3.1	3.6	0.9963
uNGAL/creatinine (μg/mmol)	1.6	1.02	0.73	0.07892^†^

*Normal values: uNGAL (median 5.2 [2.5;12.8] ng/ml), uAlb/creatinine ratio (<3.39 mg/mmol), β2-M (<0.3 mg/l)*.

* *Statistically significant value*.

† *Value aproximating statistical significance*.

**Table 5:** Kruskal–Wallis test to compare the median values of biomarkers in voided urine with respect to TTT and curve pattern (increased TTT was assumed when at least one RU on RS had TTT>3 min and obstructive curve when evacuation of radionuclide was classified as o'Reillys's B or D curve type).

***N*** **= 40 pts**	**uAlb (mg/l)**	**uAlb/crea (mg/mmol)**	**uNGAL (ng/ml)**	**uNGAL/crea (μg/ml)**	**β2-M (mg/l)**	**β2-M/crea (mg/mmol)**
**Measures**						
TTT < 3 obs^*^+ (14 pts^†^)	7.5	1.78	3.5	0.94	0.05	0.02
TTT > 3 with obs+ (20 pts)	3.5	3.22	3.1	1.81	0.05	0.05
TTT < 3 obs- (6 pts)	6.5	1.94	2	0.31	0.04	0.01
*p* value	0.4885	0.5188	0.2917	0.05878	0.4457	0.0009714^*^

* *Obstruction*,

† *Patients. There were no patients with TTT > 3 min and no obstructive curve*.

## Discussion

Early diagnosis and management of obstructive uropathy seems to be the best kidney-preserving option. Currently used diagnostic modalities to justify the final decision are often complex, invasive, and time-consuming especially including serial appointments. Numerous urine biomarkers have been investigated with an effort to find the best one to predict the necessity of surgery, and even then the true surgical indication remained obscure in many studies. In some studies, uNGAL was higher and able to distinguish between patients who were treated surgically or had renal dysfunction and healthy controls. We were able to find 4 recent studies advocating uNGAL as a promising factor effectively identifying surgical cases. The median age of patients ranged from 3 to 20 months at the time of inclusion (21–24). However, small sample size and different patterns of obstruction in the compared subgroups were the drawbacks frequently. A prospective study with a significantly larger sample size of 161 patients and a difference in the median age between subgroups of more than 20 months at the time of inclusion (similar to our cohort) demonstrated that the uNGAL level was not while CA19-9 was a significant predictor of surgery (25). Another 2 pediatric studies with the median age of patients 42 months at inclusion demonstrated no significant differences of bladder uNGAL and KIM-1 between obstructed patients before surgery and healthy controls while a significant elevation of IP-10 and MCP-1 in the pyeloplasty group was present (26, 27). KIM-1 had a better performance in the diagnosis of hydronephrosis in preoperative adult studies, and the uNGAL value was dependent on leukocyturia. This interfering factor was unfortunately not excluded in our study (28). The significance of uAlb and β2-M in congenital hydronephrosis was also described in the literature. One study described retinol-binding protein, aquaporin 2, and uAlb as potential biomarkers of obstruction with a tendency to decrease after treatment (29). Summarizing from several studies, β2-M was studied as a marker of renal damage utilized from the fetal period to later childhood. The first study described the elevation of uNGAL and β2-M in children with obstructed kidneys at the time of surgery whose median age was 8 years (older than previously described studies as well as our study) (30). Another study revealed that β2-M after sampling urine from the fetal pelvis demonstrated the significantly increased concentrations of β2-M and sodium in obstructed kidney urine (31).

Our findings suggest that the APD/mid-parenchymal ratio can be a promising index on US. It demonstrated a substantial specificity of 89% and sensitivity of 67% in the prediction of surgery. When combining it with uAlb, β2-M, and uNGAL, specificity and sensitivity increased to 94 and 71%, respectively, although insignificantly. As some of the previously mentioned studies, we also did not find any significance of uNGAL alone in the diagnosis of obstruction or prediction of operation; however, the uNGAL/creatinine ratio was close to significance when compared between obstructed and non-obstructed groups with or without increased TTT (Table 4). The urine β2-M/creatinine ratio in bladder urine appeared to be the only factor which seems to have clinical significance in determining increased TTT with an obstructive curve of at least one renal unit on RS. When all patients were stratified by age below 6 years and after 6 years, we found that there was a difference in uAlb, uAlb/creatine ratio, and β2-M in voided urine between operated and non-operated patients. None of the biomarkers were significantly different in the older patient group. This issue can be discussed under the basis of potentially different mechanisms of obstruction, as older children tend to develop hydronephrosis due to aberrant vessels, which causes intermittent hydronephrosis and kidney damage and may be influenced by the incidence of Dietl's crisis, and time-dependent injury of renal function can occur when the duration of obstruction is longer (32). The cumulative APD/mid-parenchymal ratio was significantly different between operated and non-operated patients irrespective of age.

The drawbacks of our study are the relatively small sample size and the difference in age of patients and controls. The significance of biomarkers depends on the selection of study groups, whether it was stratified by the choice of surgery or abnormal RS findings. The sample of operated and non-operated patients and the sample of patients who had normal and abnormal findings on RS overlap, but they are not the same, because some of the operated patients were not tested by RS and not all patients with abnormal RS findings were operated during the follow-up because of lack of obstructive symptoms and small dilatation.

Our findings promote the use of the cumulative APD/mid-parenchymal thickness ratio combined with β2-M and uNGAL in the workup of borderline cases and justify further quantitative proteome analysis and biomarker studies developing biomarker kits for non-invasive obstructive uropathy diagnosis (14–17).

## Conclusion

The APD/mid-parenchymal thickness ratio was a significant predictor of developing obstruction with a fair cutoff value. When combined with several urine biomarkers, sensitivity and specificity may increase. The most promising predictor in our cohort was the β2-M/creatinine ratio in voided urine especially with prolonged TTT. The risk of low DRF was also better predicted when US parameters were used together with obstruction biomarkers. These findings advocate further prospective studies for the development of multivariable diagnostic protocol and support an idea to make hydronephrosis evaluation non-invasive.

## Data Availability Statement

The raw data supporting the conclusions of this article will be made available by the authors, without undue reservation.

## Ethics Statement

The studies involving human participants were reviewed and approved by Vilnius Regional Bioethics Committee. Written informed consent to participate in this study was provided by the participants' legal guardian/next of kin.

## Author Contributions

All authors listed have made a substantial, direct, and intellectual contribution to the work and approved it for publication.

## Funding

This work was supported by Vilnius University.

## Conflict of Interest

The authors declare that the research was conducted in the absence of any commercial or financial relationships that could be construed as a potential conflict of interest.

## Publisher's Note

All claims expressed in this article are solely those of the authors and do not necessarily represent those of their affiliated organizations, or those of the publisher, the editors and the reviewers. Any product that may be evaluated in this article, or claim that may be made by its manufacturer, is not guaranteed or endorsed by the publisher.
